# Spatial distribution and determinants of high-risk fertility behavior among reproductive-age women in Ethiopia

**DOI:** 10.1186/s41182-023-00506-y

**Published:** 2023-03-06

**Authors:** Fantu Mamo Aragaw, Dagmawi Chilot, Daniel Gashaneh Belay, Mehari Woldemariam Merid, Anteneh Ayelign Kibret, Adugnaw Zeleke Alem, Melaku Hunie Asratie

**Affiliations:** 1 Department of Epidemiology and Biostatistics, Institute of Public Health, College of Medicine and Health Sciences, University of Gondar, Gondar, Ethiopia; 2 Department of Human Physiology, College of Medicine and Health Sciences, University of Gondar, Gondar, Ethiopia; 3 College of Health Sciences, Center for Innovative Drug Development and Therapeutic Trials for Africa (CDT-Africa), Addis Ababa University, Addis Ababa, Ethiopia; 4 Department of Human Anatomy, College of Medicine and Health Sciences, University of Gondar, Gondar, Ethiopia; 5 Department of Women’s and Family Health, School of Midwifery, College of Medicine and Health Sciences, University of Gondar, Gondar, Ethiopia

**Keywords:** High-risk fertility behavior, EDHS, Multilevel analysis, Spatial analysis, Ethiopia

## Abstract

**Background:**

In low-and-middle-income, including Ethiopia, high-risk fertility behavior is a major public health concern. High-risk fertility behavior has an adverse influence on maternal and child health, which hampered efforts to reduce maternal and child morbidity and mortality in Ethiopia. Therefore, this study aimed to assess the spatial distribution and associated factors of high-risk fertility behavior among reproductive-age women in Ethiopia using recent nationally representative data.

**Methods:**

Secondary data analysis was done with a total weighted sample of 5865 reproductive-aged women using the latest mini EDHS 2019. The spatial distribution of high-risk fertility behavior in Ethiopia was determined using spatial analysis. Multilevel multivariable regression analysis was used to identify predictors of high-risk fertility behavior in Ethiopia.

**Results:**

The prevalence of high-risk fertility behavior among reproductive-age women in Ethiopia was 73.50% (95% CI 72.36%, 74.62%). Women with primary education [AOR = 0.44; 95%CI; 0.37, 0.52], women with secondary and above education [AOR = 0.26; 95%CI; 0.20, 0.34], being Protestant religion followers [AOR = 1.47; 95%CI; 1.15, 1.89], being Muslim religion follower [AOR = 1.56; 95%CI; 1.20, 2.01], having television [AOR = 2.06; 95%CI; 1.54, 2.76], having ANC visit [AOR = 0.78; 95%CI; 0.61, 0.99], using contraception [AOR = 0.77; 95%CI; 0.65, 0.90], living in rural areas [AOR = 1.75; 95%CI; 1.22, 2.50] were significantly associated with high-risk fertility behavior. Significant hotspots of high-risk fertility behavior were detected in Somalia, SNNPR, Tigray region, and Afar regions of Ethiopia.

**Conclusions:**

A significant proportion of women in Ethiopia engaged in high-risk fertility behavior. High-risk fertility behavior was distributed non-randomly across Ethiopian regions. Policymakers and stakeholders should design interventions that take into account the factors that predispose women to have high-risk fertility behaviors and women who reside in areas with a high proportion of high-risk fertility behaviors to reduce the consequences of high-risk fertility behaviors.

## Introduction

Maternal and child mortality is one of the major public health problems in the world, and it is particularly serious in developing countries [1]. Maternal high-risk fertility behavior is a risk factor that hinders efforts in lowering maternal and child morbidity and mortality [2–4]. High-risk fertility behavior (HRFB) remained to be a significant cause of neonatal and under-five mortality in low- and middle-income countries (LMICs) [5–8]. Several studies have revealed that high-risk fertility behavior has an adverse influence on maternal and child health [9–11]. The consequences of high-risk fertility behavior are more severe in LMICs, where healthcare is difficult to access and there is a high prevalence of unmet family planning needs [12–14].

Women's fertility behavior is measured by maternal age at delivery, birth spacing, and birth order, which have a significant impact on women's and children's well-being [12, 15]. High-risk fertility behavior constitutes too-early (< 18 years) or too-late(> 34 years) maternal age at delivery, short inter-pregnancy birth interval, and a higher number of live births of order three or higher [2, 16–19]. According to the WHO guidelines, a birth that occurred less than 24 months after a previous birth in two consecutive births was classified as having a short birth interval [20]. The age at first birth influences how many children a woman will have during her reproductive period [21], and childbirth by a very young or very old mother is associated with an increased risk of infant and child mortality [10, 22].

Like other LMICs in Ethiopia also, high-risk fertility behavior is a major public health concern causing poor birth and child outcomes [6, 13, 23]. Preterm births, intrauterine growth restriction, neonatal mortality, stillbirths, amniotic fluid embolism, chromosomal abnormalities, and low-birth-weight newborns are associated with late motherhood (> 34 years) [7, 24, 25]. Furthermore, high-risk fertility behavior in mothers increases the risk of chronic malnutrition in children [16].

**Fig. 1:**
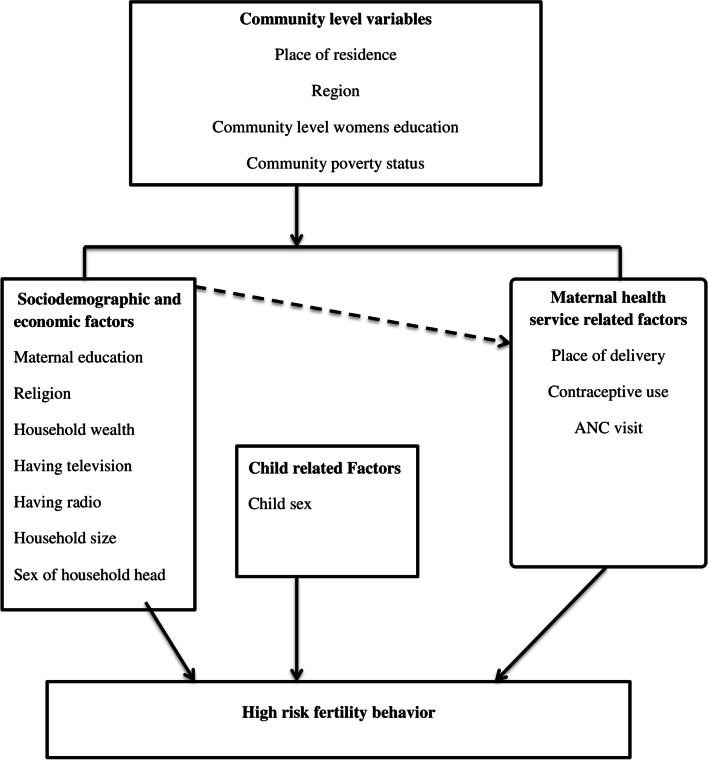
Conceptual framework of factors associated with high-risk fertility behavior developed from searching of literature

Different previous studies across the world revealed factors such as residence [4, 9, 19, 26–28], educational status [4, 12, 29, 30], partner education [29], religion [27, 31], wealth index [28], health care access challenge [32], contraceptive use [27–31, 33, 34], ANC visit [4, 32], unwanted pregnancy[31], and history of abortion [32] were associated with high-risk fertility behavior (Fig. 1).

The majority of studies about HRFB in Ethiopia focused on determining the impact of HRFB on maternal and child health outcomes [23, 35]. Even though studies about high-risk fertility behavior in Ethiopia have been conducted based on EDHS 2016, assessing the spatial variation and determinants of high-risk fertility behavior using the most recent nationally representative data (2019 EDHS) is essential for providing updated information and designing geographically focused interventions in Ethiopia. Therefore, this study aimed to answer the following research questions. What was the magnitude of high-risk fertility behavior among women of reproductive age in Ethiopia in 2019 EDHS? What are the individual and community-level factors that contribute to high-risk fertility behavior? How do the spatial patterns of high-risk fertility behavior in Ethiopia appear? The study will provide timely information to program planners and policymakers to reduce the consequences of high-risk fertility behavior.

## Methods

### Data sources, sampling procedure, and populations

For our study, we used data from the 2019 mini EDHS, which was the second EMDHS and the fifth DHS implemented in Ethiopia from March 21, 2019, to June 28, 2019. Ethiopia is located in the Horn of Africa, between 3° and 15° north latitude and 33° and 48° east longitude. The source of the population in this study was all reproductive-age women in Ethiopia within 5 years of the surveys, whereas the study population was all reproductive-age women in the selected enumeration areas within 5 years of the survey.

A two-stage cluster sampling technique was used to collect and stratify the EDHS samples. After stratifying each region into urban and rural areas, 305 (93 urban and 212 rural) clusters or enumeration areas were chosen in the first stage. The second stage of selection involved selecting a fixed number of 30 households per cluster with an equal probability of systematic selection from the newly created household listing. The detailed sampling procedure was accessible on the Measure DHS [36]. Data were obtained from the DHS website: www.dhsprogram.com after a formal request. The individual record data set was used for this analysis. A total weighted sample of 5865 reproductive-age women was included in this study.

### Variables of the study

The dependent variable was high-risk fertility which was a binary variable that was defined following the DHS guide [36]. A woman is considered to have high-risk fertility behavior if a woman is either under the age of 18 years or 34 years at the time of birth, has a birth interval of fewer than 24 months, or has the latest child of order three or higher which is coded as “1”, otherwise coded as “0” [36].

Relevant independent variables for this study were chosen based on previous literature. Individual level independent variables considered in the analysis include maternal educational level, religion, household wealth, having television, having a radio, sex of household head, ANC Visit, contraceptive use, child sex, and place of delivery. Residence, community-level poverty, community level of women's education, and region were included as community-level variables. Regions were classified into three categories; Amhara, Oromia, Tigray, and SNNP regions were categorized as a large central region; Harari, Addis Ababa, and Dire Dawa were categorized as metropolitan regions, and the others (Somali, Gambela, Afar, and Benishangul Gumuz region) were categorized as a small peripheral region [37, 38]. Because the data were not normally distributed, community poverty and literacy levels were classified as high or low, using the median value as the classification cutoff point. The poverty level in a community was categorized as high if the proportion of women in the two lowest wealth quintiles was greater than the median value, and low if the proportion was less than the median value [39]. The community level of women's education was categorized as high if the proportion of women with at least a primary level of education was greater than the median value, and low if it was less than the median value [40].

### Data management and analysis

After accessing the data, it was cleaned and coded to make it suitable for analysis. Sample weights were used to restore the survey's representativeness and obtain valid statistical estimates. STATA version 16 was used to compute both descriptive and analytic statistics. We performed a multilevel logistic regression with a random effect at the cluster level, accounting for the clustered nature of the data as well as within and between community variations, and assuming that each community has a different intercept (β0) and fixed coefficient (*β*) [41]. Variables with *p* value < 0.2 in the bi-variable analysis for both individual and community-level factors were fitted in the multivariable model, and the Adjusted Odds Ratio (AOR) with a 95% Confidence Interval (CI) in the multivariable model was used to declare statistically significant associations with the outcome variable. The goodness of fit of our model was assessed using deviance information criteria (DIC) (− 2*log-likelihood value). The Variance Inflation Factor (VIF) was utilized to test for multicollinearity among the independent variables. ArcGIS version 10.7 and SaTScan version 9.6 software were utilized for spatial and SaTScan analysis.

### Spatial analysis

Geographic variations in high-risk fertility behavior cases among EDHS clusters were assessed using spatial analysis. We computed the proportions of high-risk fertility behavior cases in the survey for each cluster and then appended the latitude and longitude coordinates of the selected EAS in the 2019 EDHS survey. The spatial autocorrelation (Global Moran's Index) statistic was utilized to determine whether high-risk fertility behavior among reproductive-age women in the study area was dispersed, clustered, or randomly distributed [42, 43] The maximum peak distance at which high-risk fertility behavior becomes more prominent was determined using incremental spatial autocorrelation [42].

Getis-Ord Gi* statistics were calculated to determine how spatial autocorrelation differs across study locations. Statistical output with a high GI* denotes a "hotspot," whereas a low GI* suggests a "cold spot" [44]. A “hotspot” area indicates a high prevalence of high-risk fertility behavior, while a “cold spot” area indicates a low prevalence of high-risk fertility behavior.

The ordinary Kriging spatial interpolation technique was used to predict areas that were not sampled based on sampled values. The spatial interpolation was performed under the assumption that spatially distributed objects are spatially correlated, and that objects that are close together are more likely to have similar properties [45, 46].

The spatial locations of statistically significant clusters for HRFB in Ethiopia were determined using Bernoulli-based model spatial scan statistics. A likelihood ratio test statistic and the *p* value were utilized to decide whether the number of observed high-risk fertility behavior within the potential cluster was significantly higher than expected.

### Parameter estimation and model building

The fixed effects were used to calculate the odds ratio with a 95% confidence interval and a *p* value of < 0.05 for the correlation between HRFB and predictor variables. The likelihood of having HRFB was modeled as follows:

$$\mathrm{log}\frac{{\pi }_{ij}}{{1-\pi }_{ij}}={\beta }_{0}+{\beta }_{1}{X}_{1ij}+{\beta }_{2}{X}_{2ij}+{\beta }_{3}{X}_{3ij}+\cdots +{\beta }_{k}{X}_{kij}+{\mathrm{u}}_{j}+\mathrm{eij}$$

where π_ij_ = is the probability of having HRFB and 1-π_ij_ represents the probability of not having HRFB. The β’s are fixed coefficients indicating a unit increase in X can cause a β unit increase in the probability of having HRFB.${X}_{1},{X}_{2}$…., ${X}_{k}$ are the individual and community-level independent variables for the ith individual in community j, respectively. The ${\beta }_{0}$ represents the intercept, which is the effect on HRFB when all independent variables are excluded. The ${\mathrm{u}}_{j}$ represents the random effect, which is the community's effect on having HRFB for the jth community, and e_ij_ represents random error at the individual level.

The random effects were determined using Intra-Class Correlation (ICC), the median odds ratio (MOR), and the proportional change in variance (PCV), which measures the variation in high-risk fertility behavior across communities or clusters. The ICC measures the differences between clusters in HRFB among women of the reproductive age group, and it is computed as ICC = $\frac{VA}{ VA+3.29}*100$, where VA = area-level variance [47–49]. When two clusters are chosen at random, the MOR represents the central value of the odd ratio between the highest and lowest risk regions. The MOR is calculated as MOR = e0.95 $\surd VA$, where VA = area level variance [41, 50].

The PCV measures how much of the total observed individual variation can be attributed to cluster differences. The PCV is calculated as; $\frac{V\mathrm{null}-VA}{V\mathrm{null}}*100$; whereas, *V*null represents the variance of the initial model, while VA represents the variance of the model with more terms [41, 50].

In multilevel analysis, four models were fitted. The first was a null model with no independent variables, which is fitted to assess the variability of HRFB in the community. The second (model I) hierarchical models contain individual-level variables, whereas the third (model II) contains community-level variables. In the fourth model (model III) both individual and community-level variables were fitted simultaneously.

### Ethical consideration

All methods were carried out following relevant guidelines of the Demographic and Health Surveys (DHS) program. Informed consent was waived from the International Review Board of Demographic and Health Surveys (DHS) program data archivists after the consent paper was submitted to the DHS Program, a letter of permission to download the data set for this study. The data set was not shared or passed on to other bodies and has maintained its confidentiality.

## Results

### Characteristics of the study participants

A total weighted sample of 5865 women aged 15–49 was included in this study. The median age of women was 29 years, with an interquartile range of 24–36 years. Most of the study participants 4236 (72.25%) live in rural areas, and more than three quarters of these (3262 (77.00%) women have HRFB. Around half of the study participants, 3025 (51.59%) have no education, and out of these, around 2555 (84.47%) participants have high-risk fertility behavior. The overall prevalence of HRFB among reproductive-age women in Ethiopia was 73.50% (95% CI 72.36%, 74.62%) (Table 1).

**Table 1:** Characteristics of the study population with high-risk fertility behavior among reproductive-age women in Ethiopia

Variables	Categories	High-risk fertility behavior		Total weighted frequency (%)
		Yes (%) *n* = 4310 (73.51)	No (%) *n* = 1553 (26.49)	
Women education status	No education	2555 (84.47)	469 (15.53)	3025 (51.59)
	Primary	1383 (65.28)	735 (34.72)	2119 (36.14)
	Secondary and higher	347 (48.32)	371 (51.68)	719 (12.27)
Religion	Orthodox	1522 (67.40)	736 (32.60)	2258 (38.51)
	Muslim	1466 (78.68)	397 (21.32)	1863 (31.78)
	Protestant	1237 (75.54)	401 (24.46)	1638 (27.94)
	Other	84 (81.60)	20 (18.40)	104 (1.77)
Household size	Less than six	1810 (58.44)	1287 (41.56)	3,098 (52.84)
	Greater than or equal to six	2499 (90.40)	265 (9.60)	2765 (47.16)
Sex of household head	Male	3874 (74.43)	1330 (25.57)	5204 (88.76)
	Female	436 (66.22)	222 (33.78)	659 (11.24)
Have radio	Yes	1203 (71.78)	473 (28.22)	1676 (28.60)
	No	3106 (74.20)	1080 (25.80)	4187 (71.40)
Have television	Yes	689 (68.01)	324 (31.99)	1014 (17.30)
	No	3620 (74.66)	1228 (25.34)	4849 (82.70)
ANC visit	Yes	3483 (70.73)	1441 (29.27)	4924 (83.98)
	No	827 (88.07)	112 (11.93)	939 (16.02)
Contraceptive use	Yes	1712 (70.52)	715 (29.48)	2428 (41.41)
	No	2598 (75.62)	837 (24.38)	3435 (58.59)
Place of delivery	Home	1498 (83.83)	289 (16.17)	1787 (30.48)
	Health facility	2812 (68.99)	1264 (31.01)	4076 (69.52)
Sex of the child	Male	2211 (79.79)	560 (20.21)	2771 (53.16)
	Female	1973 (80.81)	468 (19.19)	2,442 (46.84)
Wealth index	Poor	1767 (80.07)	439 (19.93)	2207 (37.65)
	Middle	858 (74.41)	295(25.59)	1153 (19.68)
	Rich	1684 (67.31)	818 (32.69)	2502 (42.67)
*Community level variables*				
Residence	Urban	1048 (64.43)	578 (35.57)	1627 (27.75)
	Rural	3262 (77.00)	974 (23.00)	4236 (72.25)
Community level poverty	Low	2233 (69.25)	992 (30.75)	3225 (55.01)
	High	2076 (78.72)	561 (21.28)	2638 (44.99)
Community level of women's education	Low	2194 (79.16)	577 (20.84)	2772 (47.28)
	High	2116 (68.45)	975 (31.55)	3091 (52.72)
Region	Larger central	3808 (73.74)	1356 (26.26)	5165 (88.09)
	Small peripherals	348 (79.22)	91 (20.78)	440 (7.51)
	Metropolis	153 (59.26)	105 (40.74)	258 (4.41)

### The random effect analysis result

The ICC in the null model was 0.10, which means that about 10% of the variations in HRFB among reproductive-age women were due to cluster differences, while the remaining 90% were due to individual-level factors. The PCV value, 16.66%, in the final model indicates that about 16.66% of the variation in HRFB among reproductive-age women was attributed to both individual and community-level factors. The model with the lowest deviance was selected as the best-fitted model which was model III (4775). All variables had VIF values less than 10, and the final model's mean VIF value was 1.67, indicating the absence of multi-collinearity (Table 2).

**Table 2:** Random effect analysis result

Measure of variation	Null model	Model I	Model II	Model III
VA	0.36	0.31	0.26	0.30
ICC	0.10	0.08	0.07	0.08
MOR	1.54	1.43	1.31	1.41
PCV (%)	–	13.88%	27.77%	16.66%
*Model fitness and comparison*				
Deviance	6548	4794	6495	4775
Mean VIF	–	1.33	1.55	1.67

ICC, Inter cluster correlation coefficient; MOR, Median odds ratio; PCV, proportional change in variance; VIF, Variance inflation factor

### Spatial analysis results

#### Spatial autocorrelation of high-risk fertility behavior among reproductive-age women in Ethiopia

**Fig. 2:**
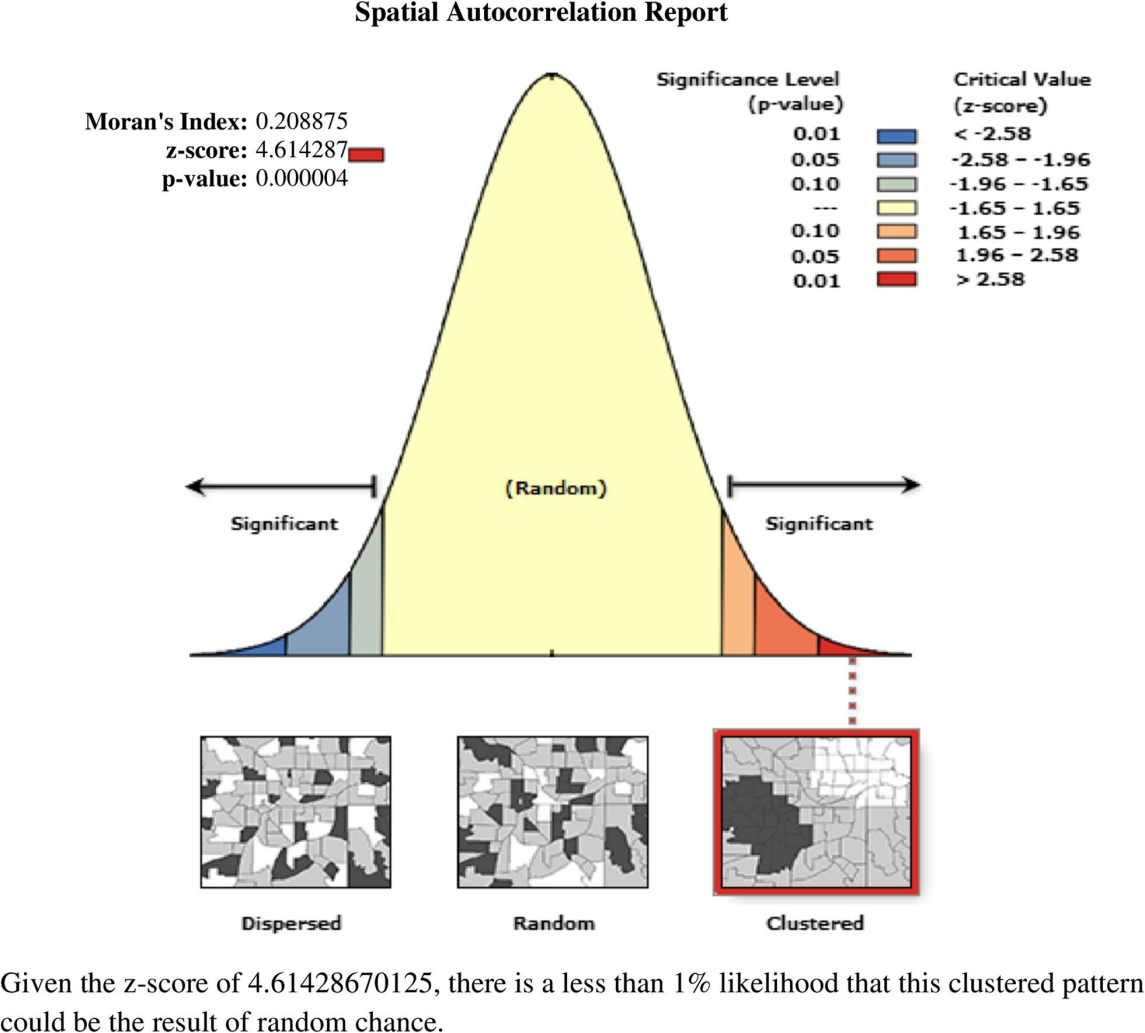
Spatial autocorrelation analysis of high-risk fertility behavior among reproductive-age women in Ethiopia, 2019 Ethiopian Demographic and Health Survey

The Global Moran's Index value was 0.208, implying that there was a significant clustering variation of HRFB among reproductive-age women and children, with a *p* < 0.001. In addition, the *Z* scores of 4.61 indicated a clustered variation of high-risk HRFB among reproductive-age women across Ethiopian regions (Fig. 2). The incremental autocorrelation result revealed statistically significant *z*-scores at a maximum distance of 275.410 km 3.25 (distances; *z*-score) for HRFB.

#### Hotspot analysis of high-risk fertility behavior among reproductive-age women in Ethiopia

**Fig. 3:**
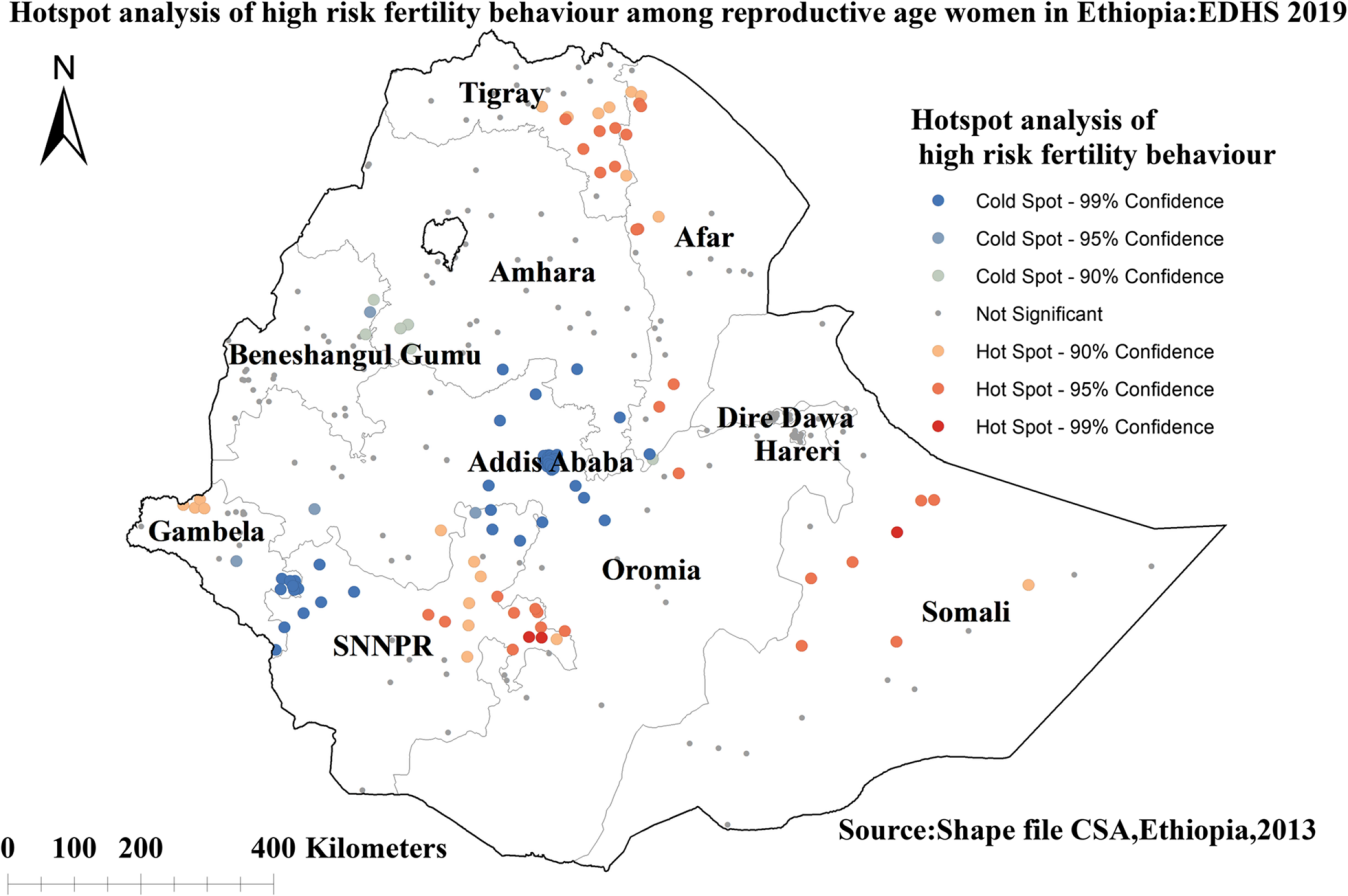
Hot spot analysis of high-risk fertility behavior among reproductive-age women in Ethiopia, 2019 Ethiopian Demographic and Health Survey

We found substantial spatial variability in the distribution of HRFB among Ethiopian women of reproductive age with significant hotspots found in Somalia, SNNPR, Tigray region, and some parts of Afar regions indicated in red color. The significant cold spots were found in Addis Ababa, the Southern border of the Amhara region, and the Northwestern part of SNNPR regions indicated in blue color (Fig. 3).

#### Spatial interpolation of high-risk fertility behavior among reproductive-age women in Ethiopia

**Fig. 4:**
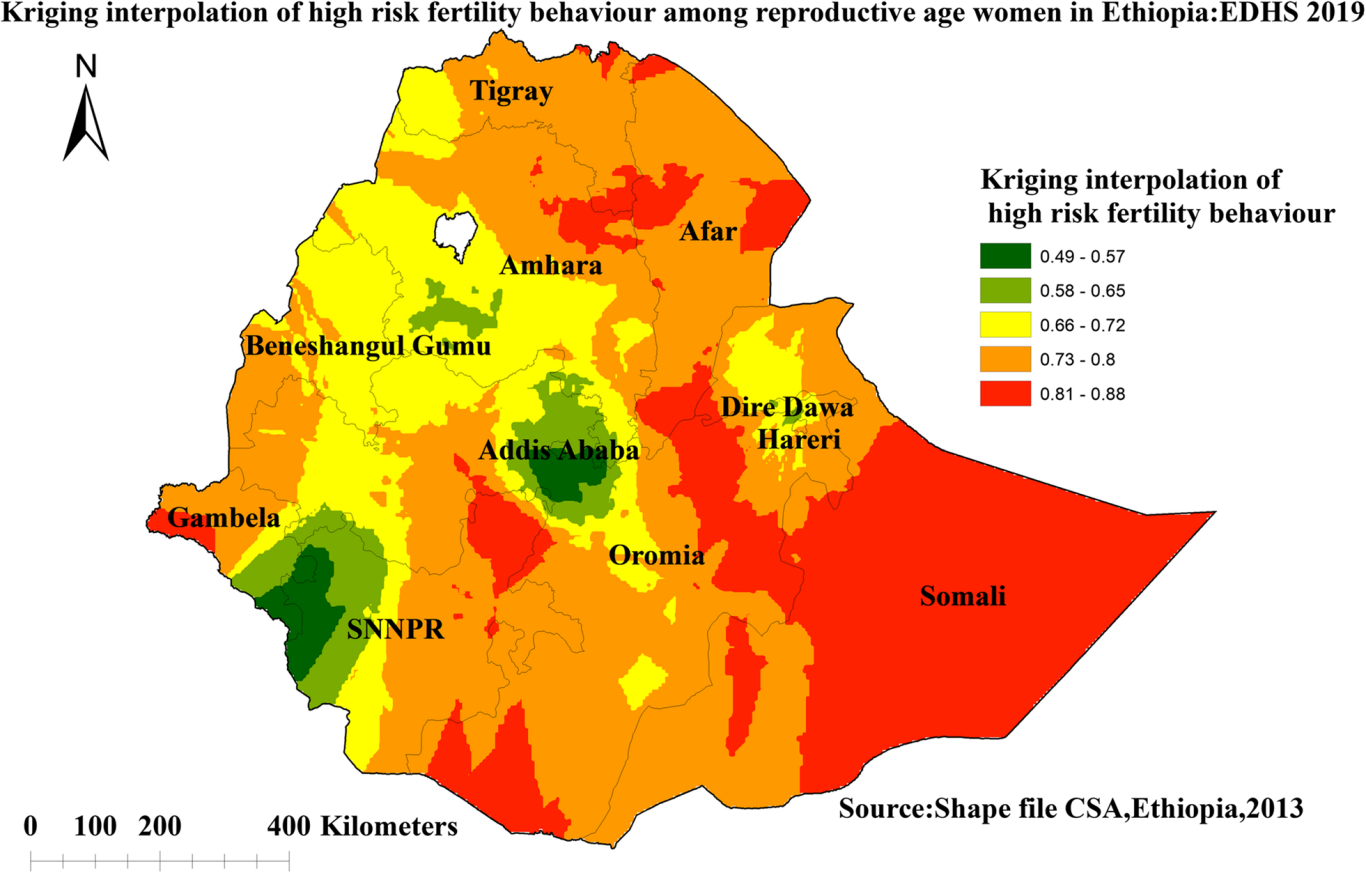
Kriging interpolation of high-risk fertility behavior among reproductive-age women in Ethiopia, 2019 Ethiopian Demographic and Health Survey

High-risk areas are indicated by red prediction zones, and women who live in such areas are more likely to have high-risk fertility behavior. The prevalence of predicted HRFB among women of reproductive age was highest in Somalia, Afar, northern SNNPR, Dire Dawa, northern Amhara, and southern Oromia regions, ranging from 81 to 88%. The lowest predicted area of HRFB was observed in Addis Ababa and the northwestern part of the SNNPR regions (Fig. 4).

#### Spatial scan statistical analysis of high-risk fertility behavior among reproductive-age women in Ethiopia

**Fig. 5:**
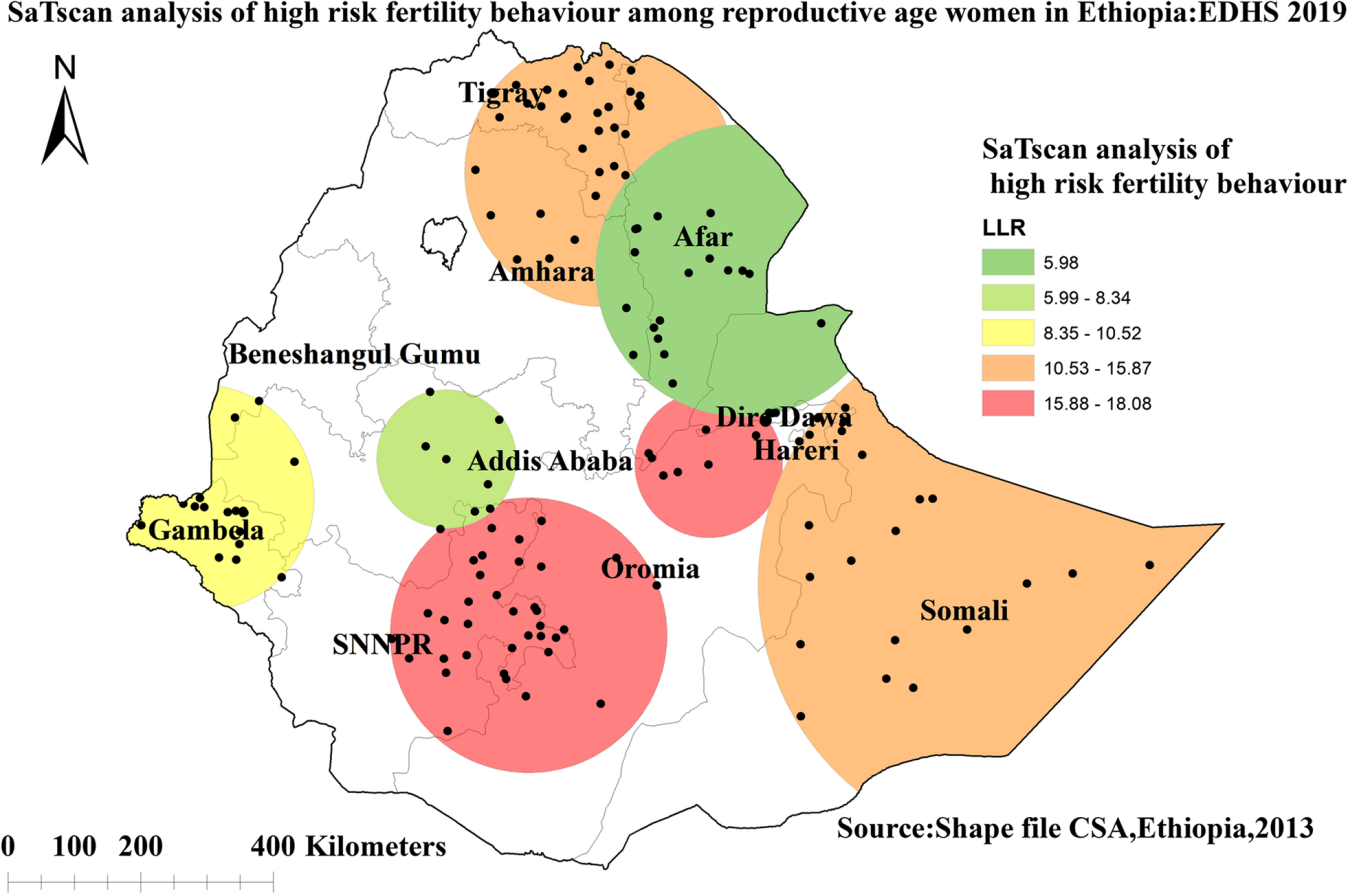
Spatial scan statistics analysis of high-risk fertility behavior among reproductive-age women in Ethiopia, 2019 Ethiopian Demographic and Health Survey

The SaTScan analysis result identified a total of 131 clusters of HRFB. The primary clusters detected in regions of Dire Dawa, Oromia, Northern SNNPR, and the southern border of Afar at 98.919051 N, 40.955418 E with a 111.22 km radius, with a relative risk of 1.25 and log-likelihood ratio (LLR) of 18.02 at a *p* value < 0.001. Women who lived within the spatial window had a 1.25 times higher chance of having high-risk fertility behavior as compared to those women outside the spatial window (Fig. 5).

### Fixed effect analysis results

According to the final model result, women's education status, religion, having television, ANC visits, contraceptive use, and residence have significant associations with HRFB among reproductive-age women in Ethiopia.

Women who were Muslims and protestant religion followers were 1.56 (AOR = 1.56; 95% CI; 1.20, 2.01), and 1.47 (AOR = 1.47; 95% CI; 1.15, 1.89) times higher odds of having HRFB, respectively, than orthodox religion follower women. Women who have primary, and (secondary and higher education) were 56% (AOR = 0.44; 95% CI; 0.37, 0.52), and 74% (AOR = 0.26; 95% CI; 0.20, 0.34) times lower odds of having HRFB than women who have no education. The odds of having HRFB among women who use contraceptives were 23% (AOR = 0.77; 95% CI; 0.65, 0.90) less compared to their counterparts. The odds of having HRFB among women who have ANC visits were 22% (AOR = 0.78; 95% CI; 0.61, 0.99) less compared to those women who have no ANC visits. The odds of having HRFB among women who have television were 2.06 (AOR = 2.06; 95% CI; 1.54, 2.76) times higher than women who have no television. Women who live in rural areas were 1.75(AOR = 1.75; 95% CI; 1.22, 2.50) times more likely to have HRFB compared to women who live in urban areas (Table 3).

**Table 3:** Multilevel multivariable analysis of factors associated with high-risk fertility behavior among reproductive-age women in Ethiopia

Variables	Categories	Null model	Model I	Model II	Model III
			AOR [95% CI]	AOR [95% CI]	AOR [95% CI]
Women education status	No education		1.00		1.00
	Primary		0.43 [0.36, 0.51]		0.44 [0.37, 0.52]***
	Secondary and higher		0.25 [0.19, 0.33]		0.26 [0.20, 0.34]***
Religion	Orthodox		1.00		1.00
	Muslim		1.56 [1.22, 1.99]		1.56 [1.20, 2.01]***
	Protestant		1.46 [1.14, 1.85]		1.47 [1.15, 1.89]**
	Other		1.89 [0.92, 3.89]		1.84 [0.89, 3.79]
Have television	No		1.00		1.00
	Yes		1.56 [1.20, 2.03]		2.06 [1.54, 2.76]***
Have radio	No		1.00		1.00
	Yes		1.14 [0.95, 1.36]		1.14 [0.95, 1.36]
Sex of household head	Male		1.00		1.00
	Female		0.82 [0.64, 1.04]		0.84 [0.66, 1.0]
ANC Visit	No		1.00		1.00
	Yes		0.77 [0.60, 0.98]		0.78 [0.61, 0.99]***
Contraceptive use	No		1.00		1.00
	Yes		0.76 [0.65, 0.89]		0.77 [0.65, 0.90]**
Sex of the child	Male		1.00		1.00
	Female		1.08 [0.93, 1.25]		1.08 [0.93, 1.25]
Wealth index	Poor		1.00		1.00
	Middle		0.94 [0.75, 1.17]		0.96 [0.76, 1.22]
	Rich		0.90 [0.71, 1.13]		1.03 [0.80, 1.32]
*Community level variables*					
Residence	Rural			1.50 [1.12, 2.02]	1.75 [1.22, 2.50]***
	Urban			1.00	1.00
Community level poverty	Low			1.00	1.00
	High			1.18 [0.92, 1.52]	1.07 [0.79, 1.45]
Community level of women's education	Low			1.00	1.00
	High			0.73 [0.57, 0.93]	0.89 [0.66, 1.19]
Region	Large central			1.00	1.00
	Small peripheral			1.21 [0.88, 1.66]	1.06 [0.70, 1.59]
	Metropolitans			0.82 [0.56, 1.99]	[0.86 [0.54, 1.36]

AOR, adjusted odds ratio; CI, confidence interval

**p value < 0.01, ***p value < 0.001

## Discussion

Nearly three fourth of women in Ethiopia had high-risk fertility behavior. The result is higher than that of a study conducted in Bangladesh [31], a pooled study in nine East African countries [32], and lower than a previous study done in Ethiopia [4]. The possible reason for the discrepancy might be differences in participants' socio-demographic characteristics and the difference in health service availability across study areas. Moreover, high birth order is the most common single risk, with nearly half of all women reporting it. The short birth interval was the least common risk, affecting nearly one-third of all women.

The spatial distribution of HRFB among reproductive-age women in Ethiopia varied greatly across regions. Significant hotspots of HRFB among reproductive-age women were detected in Somalia, SNNPR, Tigray region, and some parts of the Afar regions of Ethiopia. The SaTScan analysis result identified primary clusters found in regions of Dire Dawa, Oromia, Northern SNNPR, and the southern border of Afar. The finding was supported by previous studies that indicated living in the Somalia region are more likely to engage in HRFB, whereas women in the Amhara region are less likely to engage in HRFB [4]. The possible reason might be due to variations in health care services access across regions of Ethiopia [51]. Another possible explanation for regional differences in high-risk fertility behavior hot spot areas could be differences in demographic, cultural, and socioeconomic factors [52]. Studies also indicated that there is a low proportion of modern contraceptive utilization in the Somali, Afar, Gambela, Tigray, and Benishangul-Gumuz regions of Ethiopia [53, 54]. Furthermore, pastoralists may have limited access to health information and modern family planning services due to their high mobility and a strong commitment to cultural and religious values [55–57].

A woman's HRFB is inversely associated with her educational level. Women who have primary and secondary education had a reduced risk of HRFB than women with no formal education. This finding was consistent with the study result in Ethiopia [4, 6], Nigeria [12], and Nepal [16]. The possible explanation would be that educated women are more aware of high-risk fertility behavior than uneducated women. Another possibility is that educated women may experience a delay in becoming pregnant due to reasons related to their career path [13].

According to this study, Muslim and protestant women in Ethiopia were more likely to engage in HRFB than orthodox religious followers. This finding is in line with a study done in Bangladesh [31], India [58], and Nepal [59]. Religion has enormous social and economic relevance in most societies, and each region has beliefs about marriage, reproductive behavior, and contraceptive use [60–62]. According to studies, the use of contraception is criticized in Islamic religious teachings, causing them to have a short birth interval and a high birth order [15].

Women having ANC visits are less likely to engage in HRFB than women not having ANC visits. The study's findings are similar to those of several other study results done in Ethiopia [4]. The possible reason might be that during ANC visits, a variety of essential services are provided to promote the health of the mother and newborn including information exchange and counseling women about birth preparation and family planning options for optimal birth intervals [63].

Contraceptive users were less likely to engage in HRFB than contraceptive nonusers. The finding is similar to a study done in Ethiopia [4], Bangladesh [31], and Sub-Saharan Africa [29]. As per study results, using contraception has a significant association with lowering the burden of these high-risk births, which reduces maternal and neonatal mortality [33]. Women having television are more likely to have HRFB than women who not having a television. This finding is contradictory to previous studies in Nepal [59]. Studies indicated that exposure to mass media (radio/TV) has a significant impact on reproductive behavior [59]. The difference could be because most studies define media exposure as the frequency of watching television, listening to the radio, or reading the newspaper, whereas our study only considers possession of television, which may not be an assurance for increasing awareness of HRFB. Since we have no information on the channels and serials broadcasted on television, as well as the frequency with which women watch television, we cannot be certain that owning a television plays a positive role in having high-risk fertility behavior.

Women who live in rural areas are more likely to participate in HRFB than women who live in urban areas. This finding is in line with a study done in Ethiopia [4, 6], and East African countries [32]. The possible explanation is that women in rural areas are severely underserved in terms of maternal health services, and the majority of women in rural areas may be uneducated, causing them to be less aware of the problems associated with high-risk fertility behavior.

The study's main strength was that it used nationally representative data with a large sample size and an appropriate statistical approach that took into account the data's hierarchical nature. However, this study had limitations in that the cross-sectional nature of the data makes it impossible to infer causality between the independent and dependent variables. Since this survey relies on respondents' self-report, there may be a chance of recall bias.

## Conclusion

A significant proportion of women in Ethiopia engaged in high-risk fertility behavior. High-risk fertility behavior was distributed non-randomly across Ethiopian regions. Policymakers and stakeholders should design interventions that take into account the factors that predispose women to have high-risk fertility behaviors and women who reside in areas with a high proportion of high-risk fertility behaviors to reduce the consequences of high-risk fertility behaviors.

## Data Availability

The data sets used during the current study are available from the corresponding author upon reasonable request.

## Abbreviations

ANC: Antenatal care
AOR: Adjusted Odds Ratio
CI: Confidence Interval
COR: Crude odds ratio
DHS: Demographic and Health Survey
EAs: Enumeration Areas
EDHS: Ethiopian Demographic and Health Survey
GIS: Geographic Information System
HRFB: High-risk fertility behavior
ICC: Intra-class Correlation
MOR: Median Odd Ratio
PCV: Proportional Change in Variance
SNNP: Southern nation nationalities of people
VIF: Variance inflation factor
